# 紫杉醇脂质体单药与紫杉醇脂质体联合奥沙利铂一线治疗老年晚期非小细胞肺癌的随机对照研究

**DOI:** 10.3779/j.issn.1009-3419.2012.02.04

**Published:** 2012-02-20

**Authors:** 晓梅 曾, 之曦 李, 梅 侯

**Affiliations:** 610041 成都，四川大学华西医院肿瘤中心 Cancer Center, West China Hospital, Sichuan University, Chengdu 610041, China

**Keywords:** 紫杉醇脂质体, 奥沙利铂, 肺肿瘤, 老年, Paclitaxel liposome, Oxaliplatin, Lung neoplasms, Aged

## Abstract

**背景与目的:**

目前推荐第三代药物单药治疗老年晚期非小细胞肺癌（non-small cell lung cancer, NSCLC），本研究旨在比较紫杉醇脂质体与紫杉醇脂质体联合奥沙利铂一线治疗老年晚期NSCLC的临床疗效及毒副作用。

**方法:**

2008年7月-2010年8月未经过治疗的经病理学或细胞学确诊的老年晚期NSCLC患者69例随机分成紫杉醇脂质体单药组（35例）和紫杉醇脂质体联合奥沙利铂组（34例），单药组给予紫杉醇脂质体135 mg/m^2^ d1；联合组给予紫杉醇脂质体135 mg/m^2^ d1+奥沙利铂125 mg/m^2^ d1，每21天重复，至少治疗2个周期，评价疗效和不良反应。

**结果:**

单药组与联合组相比，治疗有效率（22.9% *vs* 35.3%, *P*=0.297）、疾病控制率（60.0% *vs* 70.6%, *P*=0.450）和1年生存率（28.6% *vs* 41.2%, *P*=0.724）差异均无统计学意义，联合组的无疾病进展生存期（progression free survival, PFS）较单药组延长1.5个月（5.0个月 *vs* 3.5个月，*P*=0.024）。在毒副作用方面，两组白细胞减少（*P*=0.808）、血小板减少（*P*＞0.999）、贫血（*P*=0.477）、恶心和呕吐的发生率（*P*=0.777）相当；两组发生神经毒性的患者分别为33例和3例（97.1% *vs* 8.6%, *P*＜0.001），但均为Ⅰ度-Ⅱ度。

**结论:**

紫杉醇脂质体联合奥沙利铂用于一线治疗老年晚期NSCLC疗效略优于紫杉醇脂质体单药，能延长患者的PFS，临床应用安全性好。

## Abstract (en)

**Background and objective:**

The third generation single-agent drug has been recommended as a first-line chemotherapy for elderly patients with advanced non-small cell lung cancer (NSCLC). The aim of the current radomized trial is to compare the clinical efficacy and toxicities of single-agent paclitaxel liposome versus paclitaxel liposome plus oxaliplatin as a first-line chemotherapy for elderly patients.

**Methods:**

Sixty-nine advanced NSCLC patients from July 2008 to August 2010, confirmed with pathology or cytology and had never received treatment, were randomly divided into two groups. The first group was given 135 mg/m^2^ of single-agent paclitaxel liposome on day 1 of each cycle. The second group was given 135 mg/m^2^ paclitaxel liposome plus 125 mg/m^2^ oxaliplatin on day 1 of each cycle. One cycle is composed of 21 days. Efficacy and toxicities could be evaluated after two or more cycles.

**Results:**

No statistical differences were observed between the two groups in terms of efficacy (22.9% *vs* 35.3%, *P*=0.297), disease control rate (60.0% *vs* 70.6%, *P*=0.450), and 1-year survival rate (28.6% *vs* 41.2%, *P*=0.724). However, the group treated with paclitaxel liposome plus oxaliplatin had longer progression free survival (PFS) (5.0 months *vs* 3.5 months, *P*=0.024). In addition, the toxicities that occurred in the two groups were similar including leukocytopenia (*P*=0.808), thrombocytopenia (*P* > 0.999), anemia (*P*=0.477), and nausea/vomiting (*P*=0.777). The number of neurotoxicity that occurred in the two groups were 33 and 3 (97.1% *vs* 8.6%, *P* < 0.001), respectively. However, all were grade Ⅰ-Ⅱ.

**Conclusion:**

The clinical efficacy of paclitaxel liposome plus oxaliplatin as a first-line chemotherapy for elderly patients with advanced NSCLC is more better than that of the single-agent paclitaxel liposome. It prolongs PFS and is safe for clinical use.

近年来肺癌已成为全球发病率和死亡率之首的恶性肿瘤。老年人的非小细胞肺癌（non-small cell lung cancer, NSCLC）发病率高，50%以上发生在65岁以上的老人，70岁以上的患者占30%-40%^[1]^，并且70%以上的患者就诊时已处于局部晚期或晚期，因此临床多采取以化疗为主的综合治疗。老年晚期NSCLC患者预期寿命短，并常合并心脑血管等基础疾病，并且免疫力低下，骨髓造血功能减弱，对化疗的耐受性差，因此目前大多推荐第三代药物单药化疗^[2]^。但近年有研究^[3]^发现老年晚期NSCLC患者可以耐受含铂类双药化疗且疗效更好。紫杉醇联合铂类化疗已经被公认为NSCLC的标准一线化疗方案之一，而紫杉醇脂质体具有更高的安全性，已经被国家SFDA批准用于晚期NSCLC的一线治疗。临床研究^[4]^证实了紫杉醇脂质体治疗老年晚期NSCLC疗效及安全性可靠。在铂类中，奥沙利铂做为第三代铂类药物，具有比顺铂和卡铂更低的血液学毒性、消化道毒性及肾功能损害^[5, 6]^。由于国外紫杉醇脂质体未上市，国内此双药联合方案治疗老年晚期NSCLC的研究鲜有报道，因此本研究旨在比较紫杉醇脂质体与紫杉醇脂质体联合奥沙利铂一线治疗老年晚期NSCLC的疗效及毒副作用。

## 1 资料与方法

### 1.1 入组标准及排除标准

入组标准：经组织学或细胞学明确的Ⅲb期/Ⅳ期未经过治疗的NSCLC，年龄≥70岁，有可评价的临床靶病灶，ECOG（Eastern Cooperative Oncology Group）体能状况评分（performance status, PS）为0分-2分，血液学（白细胞计数≥4×10^9^/L，中性粒细胞绝对值≥2×10^9^/L，血红蛋白≥80 g/L，血小板计数≥100×10^9^/L）、肝肾功和心电图等检查正常，无急性感染和重要内脏功能不全表现，预计生存期＞3个月。排除标准：局部晚期患者；出现脑转移；合并其它恶性肿瘤；合并严重的心血管疾病和控制不佳的糖尿病；接受过化疗、放疗、免疫及分子靶向治疗者。病例通过SPSS 17.0软件自带随机程序随机分组，本研究无研究基金资助，但研究用药被列入医保范畴，在入组时患者被详细告知治疗方案以及费用的相关事宜，并签署知情同意书。

### 1.2 临床资料

2008年7月-2010年8月共69例患者入组本研究，其中男性43例，女性26例，年龄71岁-83岁，中位年龄为74岁。经过随机分组，接受紫杉醇脂质体联合奥沙利铂双药化疗的联合组有34例，其中Ⅲb期16例，Ⅳ期18例；鳞癌14例，腺癌19例，其它类型1例。接受紫杉醇单药化疗的单药组有35例，其中Ⅲb期13例，Ⅳ期22例；鳞癌16例，腺癌19例。两组基线特点（男女比例、中位年龄、PS评分、疾病分期、组织学类型、吸烟等）无明显差异。患者病例特征见表 1。

**1:** 入组患者的临床特征 Clinical characteristics of patients included in this study

Characteristic	*n*	Paclitaxel liposome (*n*=35)	Paclitaxel liposome plus oxaliplatin (*n*=34)	*P*
Gender				0.624
Male	43	23	20	
Female	26	12	14	
Age（year）				0.561
Median	-	75	74	
Range	-	70-83	70-81	
ECOG performance status				0.265
0-1	53	29	24	
2	16	6	10	
Histological type				0.379
Adenocarcinoma	30	16	14	
Squamous cell carcinoma	38	19	19	
Others	1	0	1	
TNM stage				0.469
Ⅲb	29	13	16	
Ⅳ	40	22	18	
Smoking history				0.811
Smoke	36	19	17	
Non-smoke	33	16	17	

### 1.3 治疗方法

单药组给予紫杉醇脂质体135 mg/m^2^（南京思科药业有限公司）第1天静脉注射，每21天重复；联合组给予紫杉醇脂质体135 mg/m^2^+奥沙利铂125 mg/m^2^（江苏恒瑞医药股份有限公司）第1天静脉滴注，每21天重复。用紫杉醇脂质体前30 min预处理：给予地塞米松10 mg静脉注射，苯海拉明40 mg肌肉注射。至少接受2个周期化疗，每个周期记录毒副反应，2个周期以上行疗效评价。在治疗过程中发生疾病进展或出现严重不良反应时停止研究治疗。

### 1.4 疗效评价

疗效根据RECIST标准分为完全缓解（complete remission, CR）、部分缓解（partial remission, PR）、稳定（stable disease, SD）和进展（progressive disease, PD），以CR+PR占总病例数的比率计算有效率（response rate, RR），以CR+PR+SD计算疾病控制率（disease control rate, DCR）。不良反应分度按WHO毒性反应评定标准分为0度-Ⅳ度。

### 1.5 随访及统计学方法

所有患者在化疗疗程结束后安排每3个月进行门诊随访，随访内容包括患者生存情况、疾病进展情况及治疗情况等。如有病情变化或研究者认为需要进行相关复查时则随时安排检查，影像学检查由指定的影像专科医师独立完成，研究者无法干涉，疗效评价按照RECIST标准。随访截止于2011年8月。主要观察终点是无疾病进展生存期（progression free survival, PFS）和安全性。所有数据用SPSS 17.0统计包处理。率的比较采用χ^2^检验，年龄比较采用秩和检验，PFS分析采用*Kaplan Meier*法，差异性分析采用*Log*-*rank*检验。*P*＜0.05为差异有统计学意义。

## 2 结果

### 2.1 临床疗效

所有患者均完成至少2个周期化疗，无患者因治疗相关不良事件影响后续治疗。单药组共完成124个周期治疗，17例（48.6%）患者完成至少4个周期化疗；联合组共完成136个周期治疗，24例（64.7%）患者完成至少4个周期化疗。联合组完成治疗情况优于单药组。

2个周期化疗后疗效评价，单药组35例中CR 0例，PR 8例，SD 13例，RR为22.9%，DCR为60.0%；联合组34例中CR 0例，PR 12例，SD 12例，RR为35.3%，DCR为70.6%，两组相比有效率（22.9% *vs* 35.3%, *P*=0.297）和疾病控制率（60.0% *vs* 70.6%, *P*=0.450）差异无统计学意义（表 2）。

**2:** 紫杉醇脂质体和紫杉醇脂质体联合奥沙利铂的疗效评定 The efficacy evaluation of Paclitaxel liposome group and Paclitaxel liposome plus oxaliplatin group

Items	Paclitaxel liposome (*n*=35)	Paclitaxel liposome plus oxaliplatin (*n*=34)	*P*
CR (*n*)	0	0	-
PR (*n*)	8	12	-
SD (*n*)	13	12	-
PD (*n*)	14	10	-
RR	22.9%	35.3%	0.297
DCR	60.0%	70.6%	0.450
PFS (month)	3.5	5	0.024
1-year survival rate	28.6%	41.2%	0.724
CR: complete remission; PR: partial remission; SD: stable disease; PD: progressive disease; RR: response rate; DCR: disease controlled rate; PFS: progression free survival.			

单药组的中位PFS为3.5个月（95%CI: 2.4-4.9），1年生存率为28.6%（10/35）；联合组的中位PFS为5.0个月（95%CI: 2.7-7.3），1年生存率为41.2%（14/34）。两组相比，PFS差异有统计学意义（*P*=0.024）（图 1），1年生存率差异无统计学意义（*P*=0.724）。

**1:**
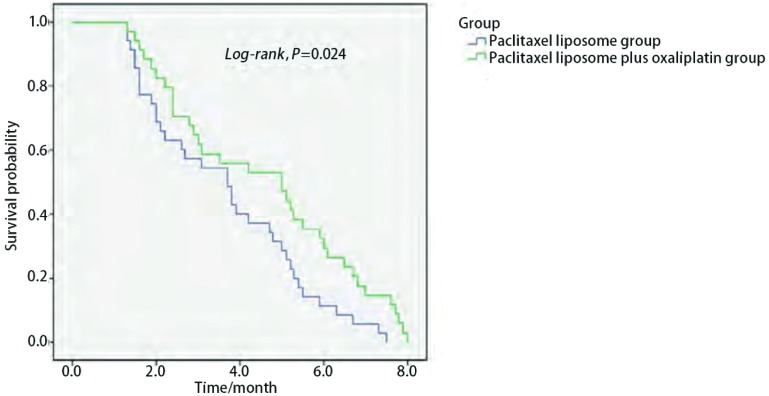
紫杉醇脂质体组与紫杉醇脂质体联合奥沙利铂组无疾病进展期比较 Comparison of progression free survival between paclitaxel liposome group and paclitaxel liposome plus oxaliplatin group

### 2.2 毒性反应

所有患者毒副反应均可评价。两组最常见的毒性反应是骨髓抑制，多为Ⅰ度-Ⅱ度，出现Ⅲ度白细胞降低在单药组合和联合组分别为4例（4/35）和3例（3/34），均无Ⅳ度出现，两组的白细胞降低(42.9% *vs* 38.2%, *P*=0.808)，贫血（8.6% *vs* 14.7%, *P*=0.477）及血小板降低发生率（11.4% *vs* 11.8%, *P*＞0.999）相比，差异均无统计学意义。其次为消化道反应，两组发生Ⅰ度-Ⅱ度恶心呕吐的患者分别为7例和8例，差异无统计学意义（20.0% *vs* 23.5%, *P*=0.777），无Ⅲ度-Ⅳ度毒性反应发生。联合组末梢神经毒性发生率明显高于单药组，联合组有33例，单药组有3例（97.1% *vs* 8.6%, *P*＜0.001），但均为轻度，给予对症处理后并不影响后续治疗。其它非血液学毒性（肝肾功损害、肌肉关节痛）均较少发生（表 3）。

**3:** 紫杉醇脂质体和紫杉醇脂质体联合奥沙利铂的毒副作用 Toxicities between paclitaxel liposome group and paclitaxel liposome plus oxaliplatin group

Toxicities	Paclitaxel liposome group (*n*=35)					Paclitaxel liposome plus oxaliplatin group (*n*=34)				*P*
	Ⅰ	Ⅱ	Ⅲ	Ⅳ		Ⅰ	Ⅱ	Ⅲ	Ⅳ	
Haematological toxicities										
Leukocytopenia	5	6	4	0		6	4	3	0	0.808
Thrombocytopenia	3	1	0	0		2	2	0	0	＞0.999
Anemia	2	1	0	0		3	2	0	0	0.477
Nonhaematologicaltoxicies										
Nausea and vomiting	5	2	0	0		7	1	0	0	0.777
Arthralgia	4	1	0	0		5	0	0	0	＞0.999
Neurotoxicity	3	0	0	0		21	12	0	0	＜0.001
Dysfunction of liver	4	0	0	0		5	2	0	0	0.342
Dysfunction of kidney	1	0	0	0		0	1	0	0	＞0.999

## 3 讨论

随着全球人口老龄化趋势加重，老年NSCLC发病率不断升高，日益受到人们的重视。老年肺癌患者因基础代谢和各脏器功能下降，免疫力低下，且大多合并肺部疾患和心血管疾病等，使得对化疗的耐受性差，化疗相关致死率较高。虽然近年来对此特殊人群治疗的研究不断涌现，但尚无治疗老年NSCLC最佳方案。因此对这些患者的治疗方案应做更多的探索，以求更高疗效和更低毒性。

紫杉醇脂质体是卵磷脂等将紫杉醇进行包裹，去除了聚氧乙基代蓖麻油溶剂和无水乙醇，从而避免溶媒引起的过敏反应。国内临床试验证实了紫杉醇脂质体治疗NSCLC的疗效及安全性^[7]^。奥沙利铂做为第三代铂类药物发生耐药机率远远小于顺铂，其抗肿瘤效应与其他铂类相当^[8]^。有文献^[9]^报道奥沙利铂与紫杉醇联合治疗初治中晚期NSCLC有效率为34.2%。Cortinovis等^[10]^对近几年相关临床研究进行分析后认为奥沙利铂对晚期NSCLC有效，且与顺铂和卡铂相比无严重的消化道毒性、血液学毒性和肾毒性。因此紫杉醇脂质体与奥沙利铂是治疗老年晚期NSCLC患者的新选择。

本研究探索了紫杉醇脂质体与紫杉醇脂质体联合奥沙利铂一线治疗老年晚期NSCLC患者的疗效与不良反应。虽然目前流行病学定义老年为年龄≥65岁，但是有证据表明年龄＞70岁的老年患者化疗相关毒性发生率增加，WHO专家建议将此作为老龄分界点^[11]^，因此本研究和大多数肿瘤临床试验相同，将老龄界定为年龄≥70岁。针对老年患者本研究给予了紫杉醇脂质体较小剂量（135 mg/m^2^），因有文献^[12]^报道紫杉醇135 mg/m^2^与250 mg/m^2^两种剂量治疗NSCLC疗效相当，而随着剂量增大，毒性反应增大。此外紫杉醇脂质体的使用说明书推荐使用剂量为135 mg/m^2^-175 mg/m^2^，且有文献^[13]^与本研究使用相同剂量并获得满意疗效。

研究中记录了一线化疗的总周期数，若患者2个周期化疗后出现病情进展，则进入二线化疗或者分子靶向治疗，结果显示单药组的化疗总周期数少于联合组。值得说明的是，经过统计检验两组间的化疗周期数差异并无统计学意义，故研究者认为组间PFS差异主要由干预措施（化疗方案）产生。联合组的有效率略优于单药组（35.3% *vs* 22.9%, *P*=0.297），1年生存率分别为41.2%和28.6%（*P*=0.724)，联合组中位PFS长于单药组（5.0个月 *vs* 3.5个月，*P*=0.024）。CALGB临床试验^[14]^分层分析了紫杉醇单药与紫杉醇联合卡铂对老年晚期NSCLC的生存期，同样得出1年生存率（31% *vs* 35%）和中位生存时间（8个月 *vs* 5.8个月）差异并无统计学意义，但是无论是本研究还是CALGB临床试验分层分析的病例数都较少，从结果来看含铂类双药化疗似乎对患者更有获益的趋势。本研究因随访时间短，并考虑到总生存时间（overall survival, OS）受后续治疗及各种混杂因素的影响，因此未对OS进行分析，两药联合是否优于单药治疗还有待进一步观察。在毒性作用上，联合组的血液学毒性并不比单药组突出，除了末梢神经毒性，其他非血液性毒性也并没显示出统计学差异。奥沙利铂的主要不良反应就是神经毒性，本研究双药组发生率（97.6%）明显高于单药组（8.6%）（*P*＜0.001），提示奥沙利铂的神经毒性不容忽视。但试验中无Ⅲ度-Ⅳ度毒性出现，对患者治疗并没有造成影响，且大多在停药后症状减轻或消失，这也证实了奥沙利铂临床应用安全性好。

综上所述，治疗老年NSCLC紫杉醇脂质体联合奥沙利铂与紫杉醇脂质体单药组相比，疗效与PFS都略显优势，毒性反应发生率并不高于单药组，是一线治疗老年晚期NSCLC的较好方案，但需要更大样本量的研究进一步探讨。
